# Dimer photofragmentation and cation ejection dynamics in helium nanodroplets

**DOI:** 10.1039/d2cp03571e

**Published:** 2022-09-30

**Authors:** Michael Stadlhofer, Bernhard Thaler, Markus Koch

**Affiliations:** Graz University of Technology, Institute of Experimental Physics, Petersgasse 16 Graz Austria markus.koch@tugraz.at

## Abstract

We present femtosecond pump–probe photoionization experiments with indium dimers (In_2_) solvated in helium nanodroplets (He_N_). At short pump–probe time delays, where the excited In_2_* is still located inside the droplet, we surprisingly observe detachment of InHe_*n*_^+^ ions with *n* = 1 to ∼30 from the droplet. These ions indicate that fragmentation of In_2_ occurs and that the kinetic energy release enables In^+^ to overcome the attractive He_N_ potential, which typically prevents ion ejection from the droplet. We find that the transient InHe_*n*_^+^ signal reveals vibrational wave packet motion in neutral In_2_*. By correlating the InHe_*n*_^+^ signal with the corresponding photoelectrons through covariance detection, we unequivocally identify the ionization pathway leading to InHe_*n*_^+^: pump-excitation from the ground-state In_2_ creates a vibrational wave packet in In_2_*, followed by probe-ionization to the cationic ground state In_2_^+^. Subsequently, a further probe photon promotes the molecule to an excited ionic state In_2_+* of nonbonding character, leading to fragmentation and kinetic energy release. This interpretation is additionally supported by probe power- and droplet-size dependencies, as well as energetic considerations. Unambiguous assignment of the ionization path to absorption–ionization–dissociation (fragmentation of the ion) in contrast to absorption–dissociation–ionization (fragmentation of the neutral) is enabled by ion ejection and electron–ion correlation. This complementary observable for ultrafast photochemical processes inside He_N_ will be particularly valuable for more complex systems.

## Abstract 2

We present femtosecond pump–probe photoionization experiments with indium dimers (In_2_) solvated in helium nanodroplets (He_N_), and identify an ionization pathway leading to ion ejection from the droplet, enabling ion-electron correlation.

## Graphical abstract

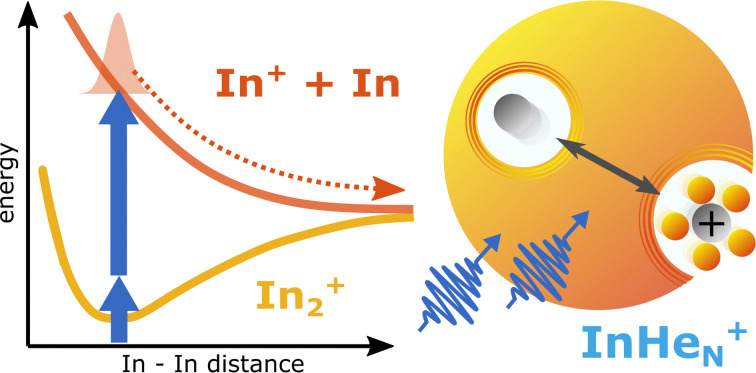

## 1 Introduction

Ultrafast photochemical reactions typically involve the concerted motion of electrons and nuclei in a non-adiabatic manner. In gas-phase, isolated from environmental influences, femtosecond pump–probe photoionization in combination with electron and/or ion detection has been developed as a versatile and powerful technique for investigating such ultrafast dynamical processes.^1–3^ While observables like the kinetic energy or angular distribution of photoelectrons primarily provide insight into electronic structure dynamics, properties of the generated ions, such as mass or kinetic energy, inform directly about processes connected to the nuclear structure, like fragmentation. In more complex situations ambiguities in the interpretation can arise, resulting from parallel relaxation pathways,^4–6^ or from the presence of several similar species, such as chromophore–solvent aggregates of different sizes.^2,7–9^ In such cases, correlating the photoelectron and -ion spectra through coincidence^10^ or covariance^11^ detection provides additional insight by revealing mass-specific electron spectra. Multiple fragmentation pathways or different species can be differentiated by analyzing the mass-specific electron spectra, which serve as a fingerprint of the species at the moment of ionization (parent *versus* fragment molecule, or size of a molecular aggregate).

Despite multifaceted developments in the field of gas-phase time-resolved spectroscopy within the last decades, some classes of molecular systems have evaded observation; examples include fragile molecules or tailor-made assemblies. The cold and controlled conditions provided by superfluid helium nanodroplets (He_N_) enable the preparation of a wide range of otherwise inaccessible systems, as demonstrated by over three decades of frequency-domain spectroscopy^12^ and mass spectrometry.^13^ Concerning time-domain studies, the opportunities of He_N_ are currently being explored and a number of photoinduced processes could be identified and characterized, including, among others, molecular alignment,^14^ coherent nuclear vibration,^15–17^ bond formation,^18^ solvent dynamics following electronic excitation,^19–22^ internal conversion,^23^ quantum beats,^24^ intermolecular energy transfer,^25^ or nanoplasma formation.^26,27^ These seminal studies have shown that, for pump–probe photoionization, the electron kinetic energy can be used as an accurate observable for processes inside He_N_ because the helium-influence on free electrons through binary collisions is sufficiently low.^28^ The influence on ions inside the droplet, in contrast, is substantially larger due to electrostrictive ion–He_N_ attraction, preventing ions from detachment from the droplet and leading to the accumulation of He atom structures around the ion with solid-like densities, called snowballs.^29,30^ This ion trapping is a severe hurdle for photochemical studies inside He_N_, as it prevents the application of ion-related detection schemes, including coincidence and covariance techniques. Ions are only available as observables if they gain sufficient kinetic energy to overcome the attractive droplet potential. Ion ejection from He_N_ has only been reported in a few experiments, where the required energy gain results either from Coulomb repulsion between ions (as applied in Coulomb explosion imaging),^14^ or from vibrational excitation by infrared light.^31,32^

Here, we present and characterize a new mechanism that facilitates the detection of ions generated inside He_N_. The mechanism builds on ionization of the excited molecules with increased laser intensity to reach a repulsive excited state, where the kinetic energy of the ion fragment is sufficient to escape the He_N_. In a first step, we show that the time-resolved ion yield can be a good observable for ultrafast intramolecular dynamics of a neutral molecule inside the droplet. In a second step, we use ion–electron correlation by covariance detection to identify the ionization pathway and the corresponding processes that lead to ion ejection from the droplet.

## 2 Experimental

We investigate indium dimers In_2_ located inside helium nanodroplets with femtosecond pump–probe photoelectron and -ion spectroscopy. As presented in previous works,^15,19^ helium droplets with a mean number of 9000 atoms (source parameters: 5 μm nozzle diameter, 15 K temperature, 40 bar stagnation pressure) are doped with, on average, two In atoms. Femtosecond laser pulses are obtained from an amplified Ti:sapphire laser system (800 nm center wavelength, 25 fs pulse duration, 4.2 mJ pulse energy, 3 kHz repetition rate). Pump pulses are tuned to the B^3^P_g_ ← X^3^P_u_ transition of In_2_ at 345 nm center wavelength (3.60 eV, 75 meV FWHM) by an optical parametric amplifier. A second harmonic of the laser fundamental at 406 nm (3.05 eV, 30 meV FWHM) provides the probe pulses. The cross correlation signal of the two pulses, defining the temporal resolution of the experiment, is estimated to be below 250 fs.

A time-of-flight spectrometer is used to detect the charge-to-mass ratio of ions and the kinetic energies of photoelectrons, applying a magnetic-bottle configuration.^33^ For correlated detection of electrons and ions, the repeller is switched voltage from −2 V to +2 kV at about 100 ns after the arrival of the laser pulses, in order to obtain both the electron and ion flight times for each laser shot.^4–6^ Covariance-mapping is applied to establish correlation between electrons and ions based on statistical fluctuations.^11^

## 3 Results

Continuing our recent studies on vibrational wave packet (WP) dynamics in In_2_ solvated inside He_N_ with time-resolved photoelectron spectroscopy,^15^ we surprisingly detect a strong InHe_*n*_^+^ ion signal at short pump–probe time delays. This ion signal is unexpected because it must result from ionization of In_2_ still located inside the droplet, where the attractive droplet potential for ions should prevent ion detection.

**Fig. 1:**
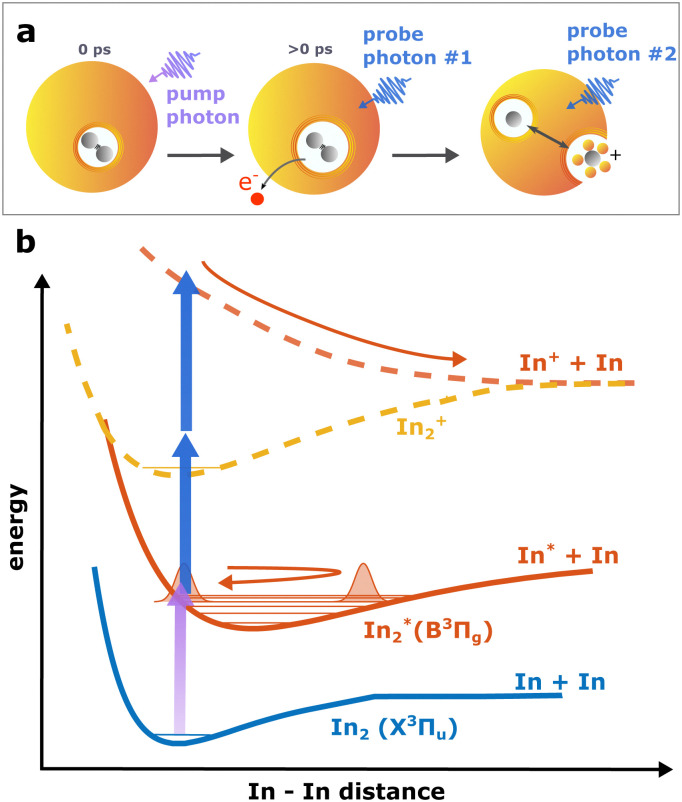
Schematic drawing of the observed process. Panel a shows the ejection process of In_2_ after photoexcitation. Following the pump excitation, the molecule is ionized by the first probe photon, and dissociates after subsequent absorption of another photon. Panel b shows a schematic of the potential energy curves of involved electronic states and the two ionization pathways: one-photon ionization leading to ground state In_2_^+^ and two-photon ionization leading to the repulsive excited state In_2_^+^* and fragmentation.

**Fig. 2:**
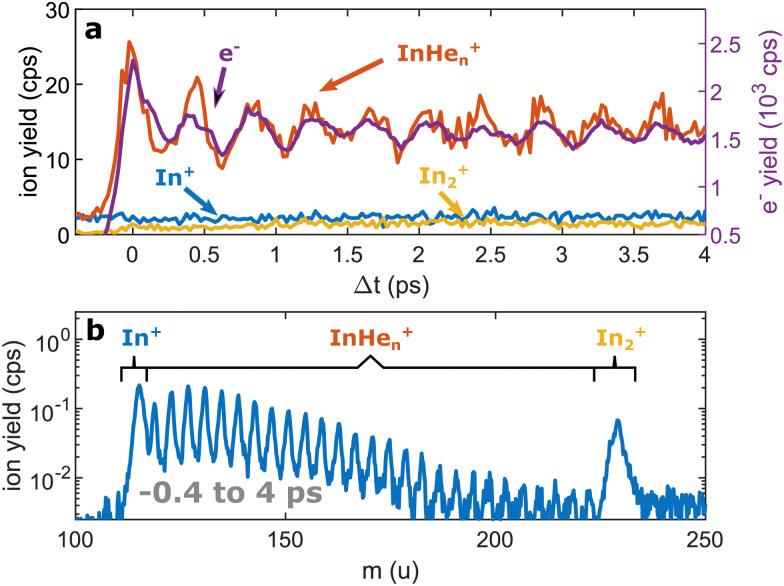
Pump–probe photoionization of In_2_ inside He_N_ at short time delays showing the initial WP oscillation. (a) Transient photo-ion signals for different mass channels, in comparison to the transient photoelectron signal.^15^ (b) Ion mass spectra obtained through integration of the transient signals up to 4 ps.

**Fig. 3:**
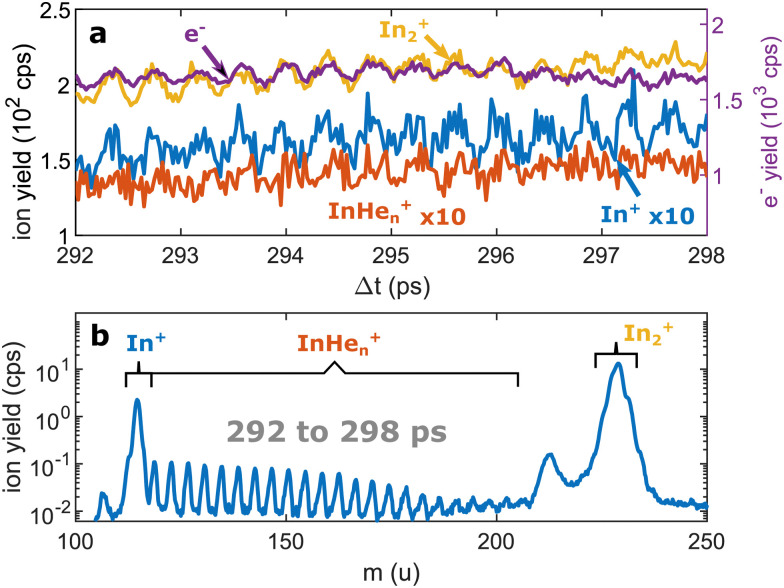
Pump–probe photoionization of In_2_ at time delays around the first full revival. (a) Transient photo-ion signals for different mass channels and transient photoelectron signal^15^ for comparison. (b) Ion mass spectra obtained through integration of the transient signals between 292 and 298 ps. The signals at 112 u and 224 u cannot be assigned.

As depicted in Fig. 1, In_2_ is excited by a pump pulse, which leads to the propagation of a WP in the excited state and results in a periodic modulation of the ionization probability.^15^ By ionizing the excited molecule with the probe pulse, the WP motion can be detected as a periodic oscillation of the photoelectron yield as a function of the time delay between the pump and probe pulses (Fig. 2a). The oscillation amplitude decays within the first few picoseconds due to dispersion within the anharmonic potential, as well as due to decoherence induced by the He environment, which could be shown to be exceptionally low compared to conventional solvents.^15^ The photoexcited In_2_* molecules are ejected from the droplet after about 50–100 ps in consequence of the repulsive In_2_*–He_N_ interaction in the excited state (see Fig. 5 and discussion below). In those bare In_2_, coherent revivals of the WP oscillation can be observed every ∼150 ps (Fig. 3a), resulting from re-focusing of the initially dispersed wave packet.

Ion signals from species inside He_N_ can usually only be expected when the ionization process happens after or close to the ejection following excitation.^34^ In contradiction to this, we observe a pronounced InHe_*n*_^+^ signal immediately after photoexcitation, way before ejection from the droplet (Fig. 2a). The InHe_*n*_^+^ ions could, in principle, originate from a single In atom, or from dissociation of a neutral or ionized In_2_ molecule. The appearance of this signal indicates that the underlying process must involve significant acceleration of the In^+^. For identifying the processes, it is important to recognize the similarity of the transient InHe_*n*_^+^ signal (red trace in Fig. 2a) and the transient electron yield (magenta trace in Fig. 2a), indicating that the InHe_*n*_^+^ ions are rooted in the WP motion of the neutral In_2_. In the following, we show that the ionization path leading to fragmentation and In^+^ detachment from the droplet proceeds *via* excitation by two probe photons into a nonbonding cationic state In_2_^+^*, as sketched in Fig. 1b. For this, we apply complementary approaches including ion mass spectra for probe-ionization inside and outside the droplet, transient ion yields, covariance-detection of electrons and ions, and ion signal dependencies on the probe-power and the droplet size.

### Ion mass spectra

We recorded ion spectra at short time-delays of the initial WP oscillation (<4 ps) and at longer time-delays in the region of the first revival of the WP oscillation (292 ps to 298 ps). The mass spectrum at short time-delays is shown in Fig. 2b: Between the In^+^ signal at 115 u and the In_2_^+^ signal at 230 u the series of peaks with mass separation of 4 u corresponds to the InHe_*n*_^+^ with *n* ranging from 1 to 27. These snowballs are likely formed during ejection of In^+^ from the droplet with additional binding of helium atoms to the charged atom, as recently also observed in Coulomb explosion imaging^35^ and photofragmentation experiments^36^ inside He_N_. The additionally present weak In_2_^+^ signal (Fig. 2b) we attribute to ionization of bare In_2,_ which might be formed by a certain fraction of small droplets contained in the size distribution, through complete He evaporation upon pickup and dimer formation. The observed In^+^ signal could come from fragmentation of these bare indium dimers or from ejection of In^+^ out of the droplet without the pickup of additional helium atoms.

Fig. 3b shows the ion masses detected at long time-delays. Both the In^+^ and In_2_^+^ signals are now dominant over InHe_*n*_^+^, indicating that they originate from ionization of bare, electronically excited In_2_*, which have been ejected from the droplet. The relative abundance of In_2_^+^ and In^+^ gives the branching ratio for ionization into the bound cationic ground state relative to ionization into the dissociative cationic state (see Fig. 1). The width of the In_2_^+^ peak appears broader than the other peaks, most probably reflecting the In_2_* kinetic energy distribution after ejection, which will be more precisely determined with velocity map imaging in a future experiment. The InHe_*n*_^+^ signal is significantly weaker but still present, which might be connected to the formation and subsequent fragmentation of In_2_*He_*n*_ exciplexes: Excited-state molecules with a number of attached helium atoms. Note that the excited Π state (Fig. 1) can be expected to support the attachment of He atoms, similar to exciplex formation of photo-excited alkali-metal atoms in p-states.^18^ Additional peaks at 112 u and 224 u could not be assigned.

### Time-resolved ion spectroscopy

**Fig. 4:**
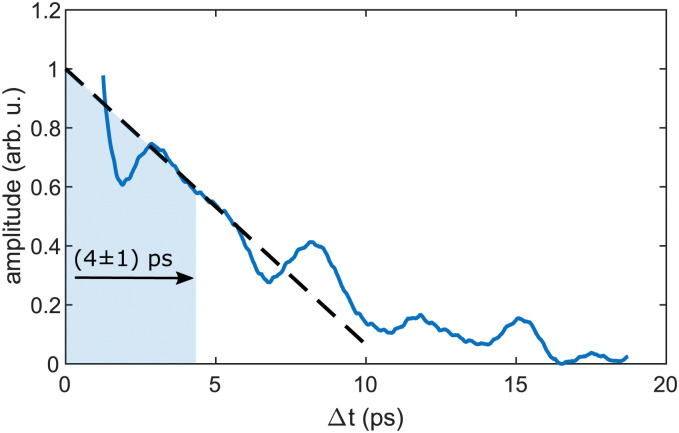
Sliding-window Fourier analysis of the transient InHe_*n*_^+^ signal at short time delays. The plot shows the transient amplitude of the periodic 2.42 THz oscillation, which is the central frequency in the spectrogram. A Hamming window of 2.5 ps width was used.

As the similarity of the transient InHe_*n*_^+^ signal and the transient photoelectron yield (Fig. 2a) strongly suggests a connection between the vibrating In_2_ and the ejected InHe_*n*_^+^ ions at early time delays, we apply sliding-window Fourier transformation for further quantification (see Fig. 4). With this method, a spectrogram of the transient InHe_*n*_^+^ signal is generated, revealing oscillation amplitudes and corresponding frequencies as a function of time. The oscillation frequency remains unchanged at 2.42 THz and the oscillation amplitude decreases with a half-life of (4 ± 1) ps. Both results are in perfect agreement with the electron transients,^15^ tracing the InHe_*n*_^+^ oscillations of solvated In_2_.

At longer pump–probe delays in the range of the first full revival around 295 ps, the In_2_^+^ signal dominates (Fig. 3a) and its oscillations again match those of the electron yield. This indicates the preservation of WP coherence during ejection from the droplet. The weaker In^+^ and InHe_*n*_^+^ signals also exhibit the same oscillation, however with much smaller amplitude.

**Fig. 5:**
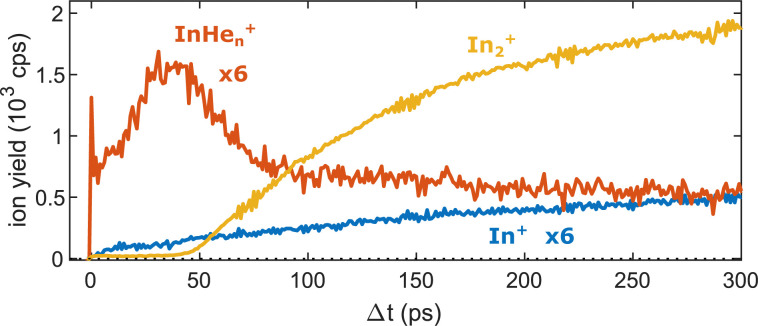
Transient ion yields of the different mass channels up to 300 ps. Note that the In^+^ and the InHe_*n*_^+^ yields are scaled up by a factor of 6.

Fig. 5 shows the progression of the InHe_*n*_^+^, In_2_^+^ and In^+^ signals over the full time window of the experiment, including the initial WP oscillations (<5 ps, Fig. 2a) and the first revival (∼295 ps, Fig. 3a). The In_2_^+^ signal reflects the characteristic ejection behavior: A pronounced onset at 50 ps with a very small ion yield before and a saturating increase up to 300 ps afterwards. The revival structure of the WP signal is also apparent in the In_2_^+^ signal: the full revival at ∼295 ps, half revival at ∼150 ps and fractional revivals in between. As this coherent signal was previously analyzed in detail,^15^ the revivals are, however, not well resolved here due to undersampling. The In^+^ transient also shows a steady increase, similar to the In_2_^+^ signal, but is significantly larger in the beginning and completely lacks a sharp onset. In contrast, the InHe_*n*_^+^ signal exhibits a very different transient behavior up to 100 ps (Fig. 5), as it is larger than both the In^+^ and In_2_^+^ signals below 50 ps and not at all monotonic. It increases promptly after the pump pulse excitation, reaching a maximum at ∼45 ps, somewhat before conventional ejection is completed (see also droplet-size dependency measurements and discussion below). Both In^+^ and InHe_*n*_^+^ seem to reach a plateau at 300 ps.

### Electron–ion correlation spectroscopy

**Fig. 6:**
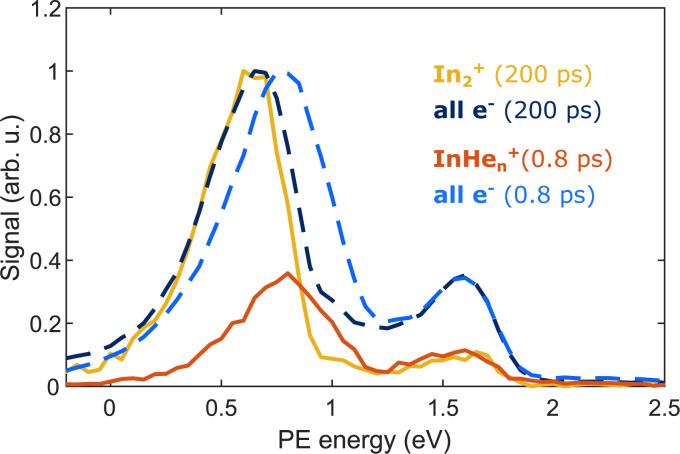
Covariance detection of electrons and ions after pump–probe ionization of In_2_ inside He_N_, showing the photoelectron spectra correlated to the questioned InHe_*n*_^+^ ions at 0.8 ps delay (red trace) and to In_2_^+^ ions at 200 ps delay (yellow trace). Additionally, the full photoelectron spectra (normalized) at the corresponding time delays are shown as dashed lines. The energy peak at 1.6 eV is presumably due to false correlation of electrons from pump-only ionization and the respective ions.

The appearance of InHe_*n*_^+^ ions upon In_2_ ionization enables us to correlate the ionization products – ions and electrons. Fig. 6 compares the electron–ion covariance spectra at short (0.8 ps) and long (200 ps) time-delays. At 200 ps delay (red trace), electrons correlated with In_2_^+^ are most prevalent with a peak at 0.65 eV in the energy spectrum, identifying the photoelectron band corresponding to In_2_* ionization after ejection from the droplet. At 0.8 ps delay (red trace), the electron signal correlated to InHe_*n*_^+^ ions peaks at slightly higher energies of 0.8 eV. The location of this peak in the spectrum is a further indication that InHe_*n*_^+^ originates from unfragmented In_2_* molecules and rules out In atoms as the source, since the photoelectron peak for ionization of excited In* atoms (5s^2^6s,^2^S) would be expected at ∼0.32 eV,^19^ and the peak for two-photon ionization of ground-state In atoms similarly at ∼0.32 eV.^37^ Additionally, photoionization of atoms produces sharper peaks.^19^ The shift to higher energies for in-droplet ionization of In_2_*, compared to bare In_2_*, is due to a lowering of the ionization potential by the He environment and possibly also indicates that the bubble expansion resulting from photoexcitation is not completed after 0.8 ps.^19^

Covariance mapping thus provides insight into the reaction pathway leading to InHe_*n*_^+^ ion ejection: The photoelectron spectrum correlated to InHe_*n*_^+^ indicates that the probe pulse ionizes In_2_* to the In_2_^+^ cationic ground state. Consequently, subsequent excitation to a repulsive In_2_^+^* state by a second probe photon must be responsible for fragmentation and ion ejection. The electrons from initial In_2_* ionization and the InHe_*n*_^+^ fragmentation products then originate from the same process and their correlation is identified through covariance detection. Note that the electron–ion covariance spectra (solid lines in Fig. 6) at 0.8 and 200 ps time delay are similar to the full electron spectra (dashed lines), with significant deviations only at the pump-only peak at 1.6 eV. This similarity shows that in this simple situation the spectra corresponding to the questioned InHe_*n*_^+^ generation inside the droplet and In_2_^+^ generation outside could be separated in time. In more complex situations with multiple parallel ionization channels, the corresponding electron spectra can only be obtained with covariance mapping.

### Probe-pulse power dependence

**Fig. 7:**
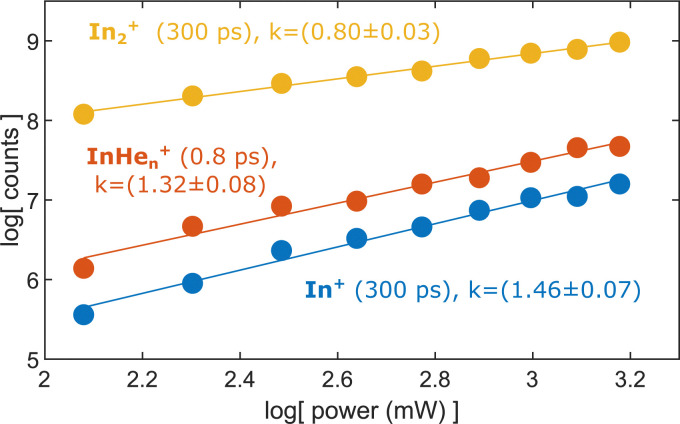
Yield of relevant ions at short and long pump–probe delays in dependence on the probe laser power.

To test the hypothesis of photoinduced dissociation of In_2_^+^ through a second probe photon, we monitor the InHe_*n*_^+^ yield at short pump–probe delays (0.8 ps) as function of the probe pulse power, in comparison to the power dependencies of In_2_^+^ and In^+^ at long delays (300 ps). Fig. 7 shows these power dependencies in a log–log plot, which allows us to determine the number of probe photons involved in the ionization process of the respective ion from the signal slope. The slopes obtained for the ion fragments, InHe_*n*_^+^ and In^+^, both exceed the value of one, suggesting that two probe photons are involved in the ionization process. In contrast, the unfragmented In_2_^+^ results from a single probe-photon process, as its slope falls below one.

This supports the assumption that at short time delays, the InHe_*n*_^+^ signal results from excitation of In_2_^+^ to a repulsive In_2_^+^* state subsequent to In_2_ ionization inside the droplet (see Fig. 1). The excited, repulsive state seems to provide sufficient kinetic energy for In^+^ to escape from the attractive droplet potential, accompanied by InHe_*n*_^+^ snowball formation. Without energy transfer of subsequent photoexcitation, ionization to the In_2_^+^ cationic ground state remains unobserved due to trapping inside the droplet.

At longer delays, after In_2_* has been ejected from the droplet, both ionization channels are observed: ionization with one probe photon to the cationic ground state leads to detection of In_2_^+^ whereas ionization with two probe photons to the excited cationic state leads to fragmentation and In^+^ detection.

### Helium droplet size dependence

**Fig. 8:**
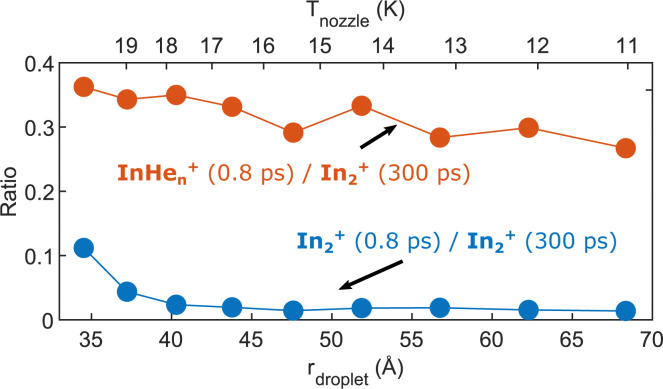
Droplet size dependence of the InHe_*n*_^+^ and In_2_^+^ yields at short pump–probe delays. Both signals are normalized to the In_2_^+^ signal at 300 ps delay.

As last step, we investigate the dependence of the InHe_*n*_^+^ and In_2_^+^ yields at 0.8 ps time delay on droplet size by variation of the nozzle temperature. To compensate for the varying He_N_ flux for different nozzle temperatures and for different pickup conditions, we normalize both signals to the In_2_^+^ signal at 300 ps, which serves as reference for the amount of ionized In_2_ molecules per laser shot. Results are shown in Fig. 8. The probability of detecting InHe_*n*_^+^ (red curve) decreases for larger droplets, while the probability of detecting In_2_^+^ (blue curve) is independent of the droplet size, at least down to very small droplets. This trend might indicate that the probability of complete droplet evaporation starts to play a role for these droplets.

## 4 Discussion

The reaction path leading to InHe_*n*_^+^ formation and ejection from the droplet can be assigned to two-photon ionization from an electronically and vibrationally excited In_2_* molecule to a dissociative cationic state. The kinetic energy release due to dissociation enables InHe_*n*_^+^ to overcome the attractive droplet potential, at least with some probability. The ejection probability seems to depend on the In_2_* location at the moment of ionization, indicated in Fig. 5 by the InHe_*n*_^+^ signal increase (red trace) within the first ∼40 ps pump–probe delay. The observed InHe_*n*_^+^ increase can be explained by pump excitation of In_2_ to a *heliophobic* state, triggering the movement of In_2_* towards the droplet surface within ∼50 ps, evidenced by the onset of the In_2_^+^ signal at this delay (yellow line in Fig. 5). Ionization of In_2_* closer to the droplet surface increases the In^+^ ejection probability, which nevertheless forms InHe_*n*_^+^ due to the attractive He interaction. This interpretation is in line with the dropletsize dependence of the InHe_*n*_^+^ signal at fixed pump–probe delay (Fig. 8, red trace): for smaller droplets InHe_*n*_^+^ ions detach from the droplet with a higher probability.

The InHe_*n*_^+^ signal decrease above 45 ps in Fig. 5 reflects the onset of In_2_* ejection, since InHe_*n*_^+^ formation requires ionization inside the droplet, whereas In_2_* ionization outside the droplet yields In_2_^+^ and apparently also fragmentation to In and In^+^. Interestingly, the InHe_*n*_^+^ signal does not decrease to zero but levels off at ∼100 ps instead, which we attribute to the ejection of neutral In_2_*He_*n*_ exciplexes followed by complete fragmentation into In and InHe_*n*_^+^ after ionization as no In_2_He_*n*_^+^ are present in the mass spectrum near 300 ps (Fig. 3).

To rationalize the proposed ion ejection mechanism, knowledge of the In_2_^+^ potential energy curves would be necessary. Since no calculated potentials are available for In_2_^+^ we instead consider the potentials of aluminum dimer cations (Al_2_^+^) in the ground and excited states,^38^ since Al_2_ and In_2_ have a similar electronic structure. Relevant potentials are shown schematically in Fig. 1. The vertical excitation energy from the ground to the lowest excited state of the cation is approximately 3.0 eV,^38^ which is in the range of our probe photon energy. With a binding energy of the cationic ground state of about 1.4 eV we estimate the total kinetic energy release to be about 1.6 eV, divided equally between both fragments. The initial velocities of In and In^+^ fragments is thus about 1160 m s^−1^. It is known, that particles with velocities above the critical Landau velocity of 56 ms^−1^ experience strong deceleration due to friction inside He_N_.^39^ This Landau velocity corresponds do 1.8 meV in the case of In, suggesting that the generated In^+^ must experience a strong drag. Furthermomre, the In^+^ binding energy to the droplet is ∼90 meV.^19^ This comparison rationalizes our interpretation: After dissociation, the initially fast In^+^ (∼1160 ms^−1^) is efficiently decelerated and can only leave the droplet if it approaches the surface with sufficient energy to escape the holding potential (∼90 meV).

## 5 Conclusion

In this paper, we identified an ionization–fragmentation channel of In_2_ molecules fully solvated inside helium nanodroplets. With pump–probe photoionization at early delay times, where the ionization takes place inside the droplet, we observe the ejection of InHe_*n*_^+^, despite the attraction of ions by He droplets. The InHe_*n*_^+^ yield shows a characteristic modulation, which exactly follows the previously observed electron yield modulation.^15^ This similarity clearly identifies the coherent vibrational WP motion in the neutral, electronically excited In_2_* as intermediate state of the ionization path. The ejection of InHe_*n*_^+^ allows us to measure the photoelectron spectrum that is correlated to these ions through covariance detection (Fig. 6). The covariance spectrum further solidifies the assumption that excited In_2_* molecules are ionized, while dissociation to In atoms before ionization seems not to be relevant. We thus conclude that the ionization path proceeds *via* ionization of the unfragmented In_2_* and fragmentation occurs in the cationic state (absorption–ionization–dissociation pathway) and not *via* fragmentation of the neutral In_2_* and ionization of In fragments (absorption–dissociation–ionization pathway). This conclusion is deduced from the similarity of the photoelectron spectra associated with InHe_*n*_^+^ at short delays and In_2_^+^ at long delays, after In_2_* has been ejected. Such unique insight is exclusively provided by correlation spectroscopy and cannot be determined unambiguously by uncorrelated photoelectron and photoion detection.^2^ Fragmentation after ionization in the cationic state is initiated by absorption of a second probe photon, as indicated by the probe laser power dependence of the InHe_*n*_^+^ yield (Fig. 7). Further insight into the fragmentation process could be gained by measuring the fragment velocities in a velocity map imaging detector, in analogy to Coulomb explosion^35^ and photofragmentation^36^ measurements. Such an experiment would be particularly interesting in view of the peculiar ion yield transient, shown Fig. 5, and in terms of energy balance investigations, to gain insight into the droplet-induced deceleration through friction.

The feasibility of electron–ion coincidence/covariance detection will be especially interesting for photochemical studies on molecular assemblies inside He_N_. The versatile techniques available for loading the droplets will provide access to ultrafast processes in novel dopant–acceptor combinations. Photoinduced processes in such assemblies, like charge transfer, often strongly depend on the cluster size.^2^ While electron–ion correlation is a prerequisite for a size-selective analysis, fragmentation after ionization in consequence of energy transfer to vibrational modes (absorption–ionization–dissociation pathway), significantly biases results obtained with bare clusters in gas phase. The stabilizing properties of He droplets, acting as a thermal bath^40^ seems promising to cool the cluster and prevent such misleading fragmentation. Correlation spectroscopy in He_N_ will potentially help to overcome ambiguities encountered by photoelectron spectroscopy of complex systems in gas phase.

## Conflicts of interest

There are no conflicts to declare.

## Supplementary Material
